# Supplementary figures and images for: In vitro formation and extended culture of highly metabolically active and contractile tissues
Source: PLoS One. 2023 Nov 1;18(11):e0293609. doi: 10.1371/journal.pone.0293609 (PMC10619834; doi:10.1371/journal.pone.0293609)

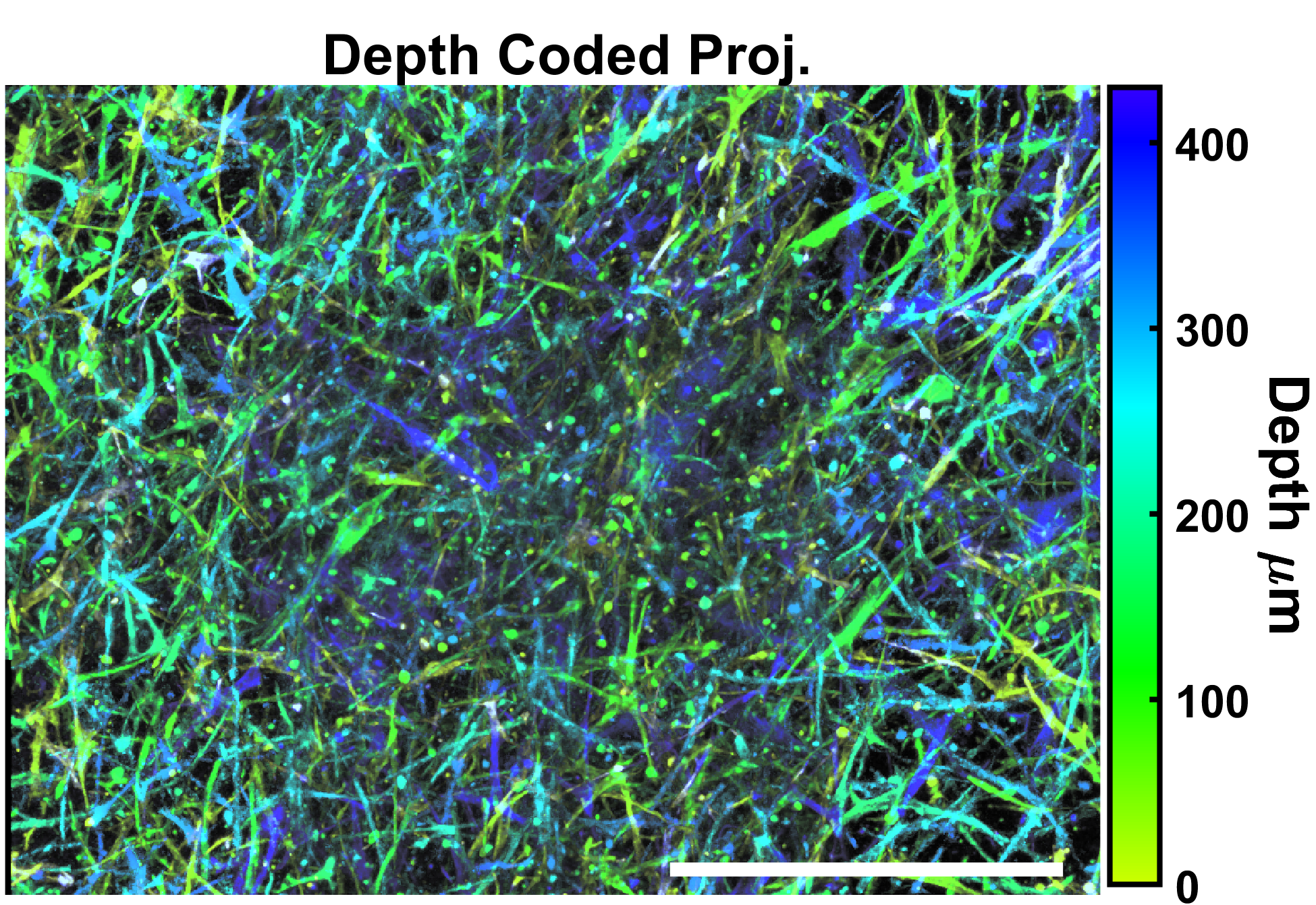

Supplement: S1 Fig — ECs and ASCs were cultured in VBAM differentiation media with VEGF (weeks 0–2) or PDGF-BB (weeks 2–4) before being fixed and stained against collagen IV (EC basement membrane marker). Shown is a depth-coded projection of a stitched tilescan demonstrating vascular networks have assembled and are present through the culture bulk (scalebar = 250 μm). (TIF) [file pone.0293609.s001.tif]

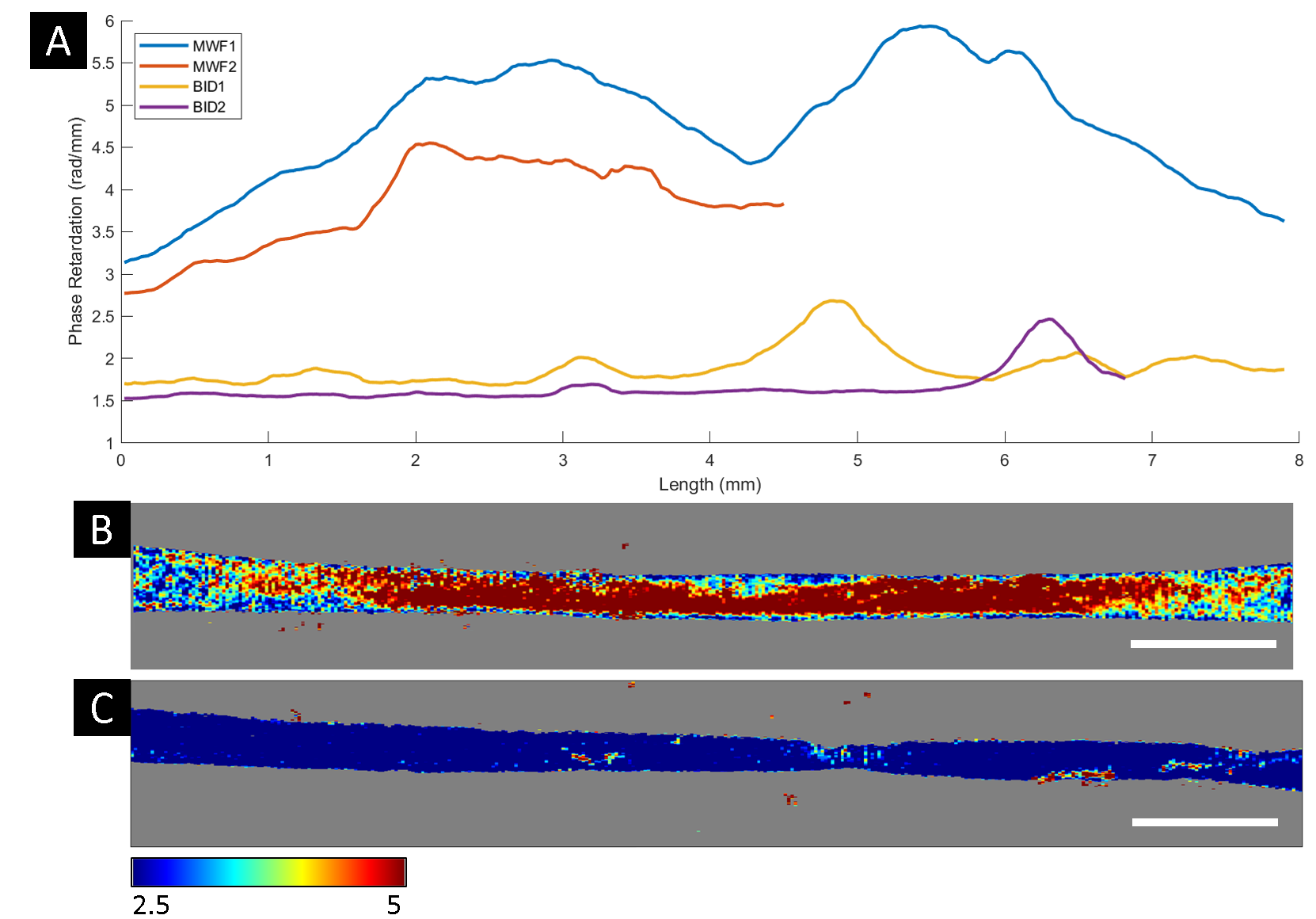

Supplement: S2 Fig — (A) The average phase retardation as a function of sample length for the MWF and B.I.D samples. The whole length of the B.I.D2 sample could not be obtained because of the pins used to fix the samples during imaging. (B-C) Representative en face phase retardation images (in rad/mm) taken from the middle of the MWF1 and BID1, respectively (scalebar = 1 mm). The MWF samples have higher phase retardation throughout the length of the samples compared to the B.I.D samples. (TIF) [file pone.0293609.s002.tif]

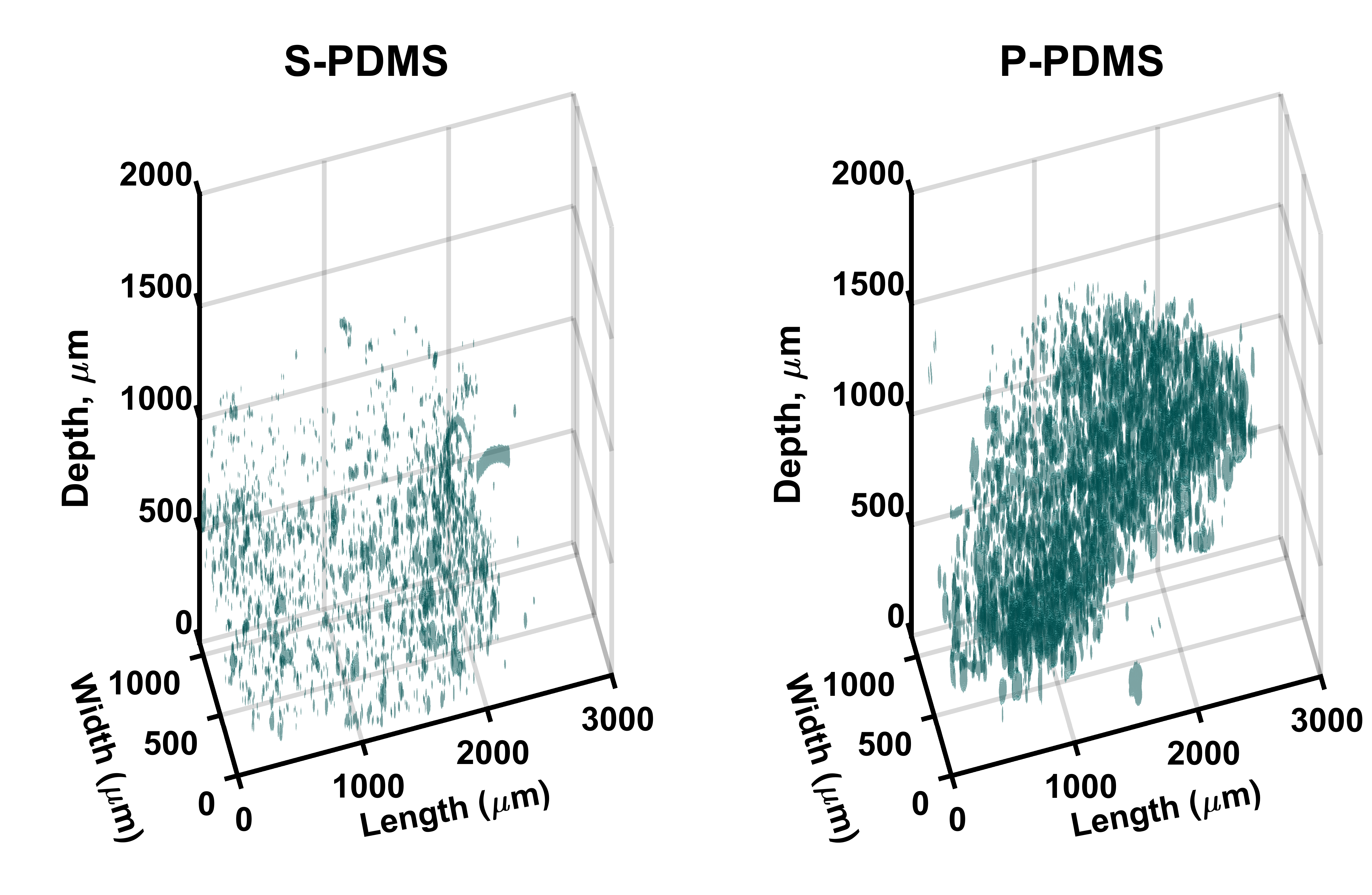

Supplement: S3 Fig — Stitched tilescans were rendered in 3D to visualize spatial distribution of nuclei. Density of P-PDMS nuclei appears increased with evidence of dense aggregate formation relative to S-PDMS. (TIF) [file pone.0293609.s003.tif]

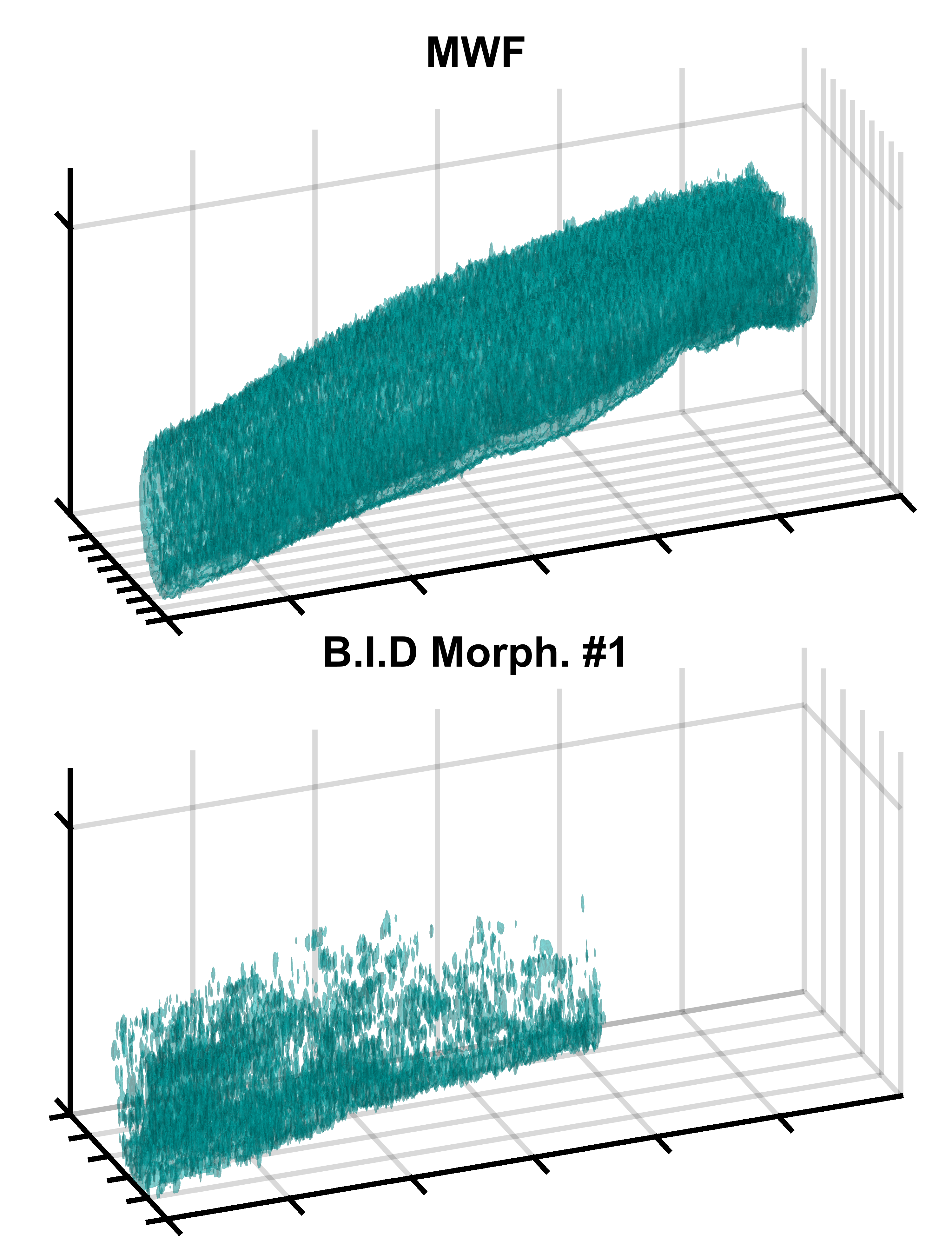

Supplement: S4 Fig — Nuclei density is increased with consistent organization globally in MWF VBAMs. (TIF) [file pone.0293609.s004.tif]

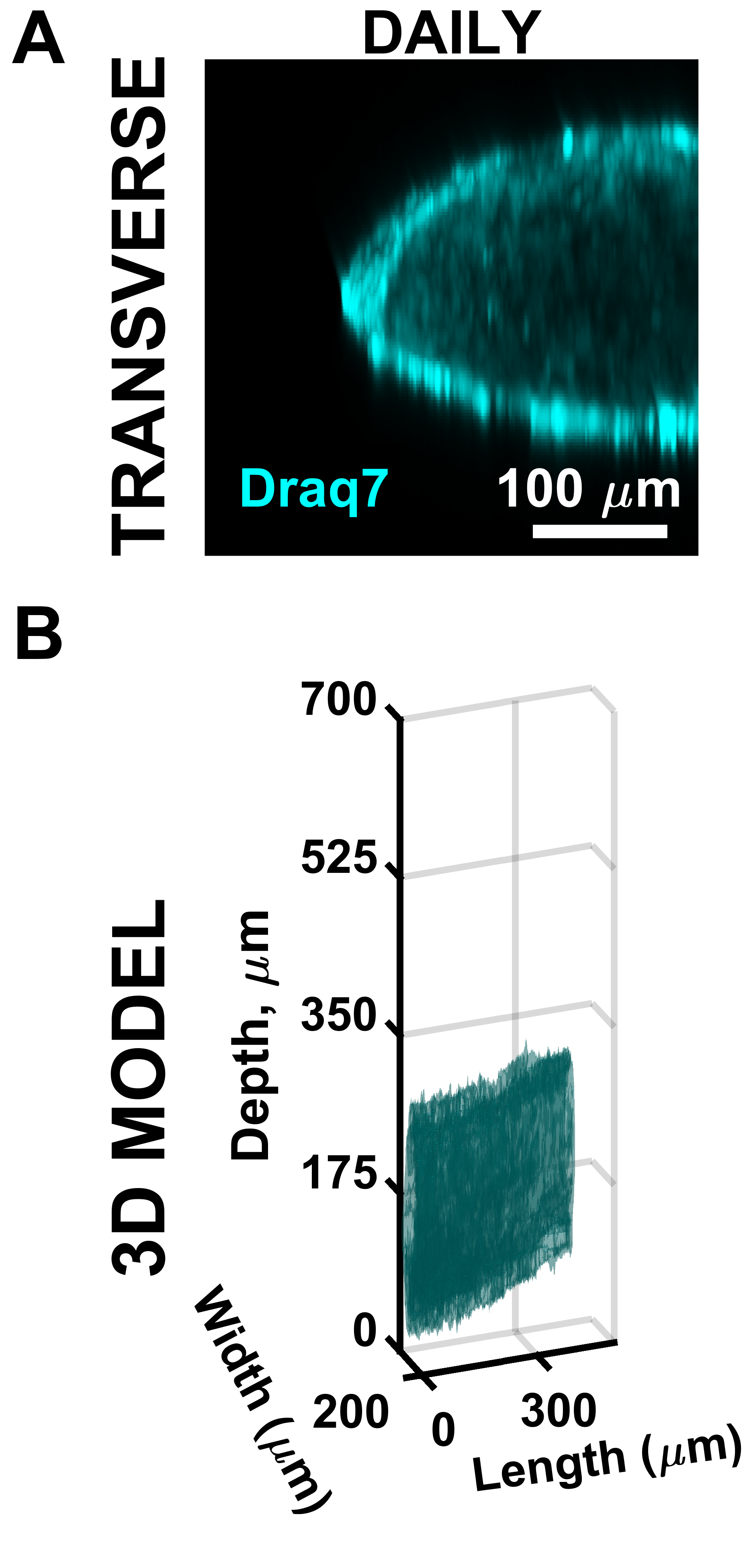

Supplement: S5 Fig — (A) Representative transverse projection of VBAM nuclei after 5 weeks of daily media changes show similar nuclei density to MWF samples. (B) 3D rendering of segmented nuclei of daily fed VBAM indicate similar spatial organization of the nuclei to MWF samples. (TIF) [file pone.0293609.s005.tif]
