# Supplementary material for: In vitro formation and extended culture of highly metabolically active and contractile tissues
Source: PLoS One. 2023 Nov 1;18(11):e0293609. doi: 10.1371/journal.pone.0293609 (PMC10619834; doi:10.1371/journal.pone.0293609)
Supplement: S1 Table — Composition of media blends that are used for cell culture. (DOCX) [file pone.0293609.s006.docx]

| **Media** | **Source** | **Components** |
| --- | --- | --- |
| IMR90 Fibroblast cell line | Corning, Manassas VA  #10-090-CV | DMEM/F12 50:50 |
|  | Corning  #35-010-CV | 10% Fetal Bovine Serum (FBS) |
|  | Genesee Scientific, El Cajon, CA  #25-512 | 1% Penicillin/Streptomycin (P/S) |
| MDCK Epithelial cell line | Corning  #10-013-CM | DMEM HG |
|  | * | 3% FBS |
|  | * | 1% P/S |
|  | VWR Life Sciences, Radnor, PA  #IC0219504391 | Amphotericin B [250 µg/mL] |
| HMEC-1 Endothelial cell line | Gen Depot, Baker, TX  #CM034-050 | MCDB131 Base medium |
|  | * | 10% FBS |
|  | * | 1% P/S |
|  | Fisher Bioreagents, Waltham, MA  #BP379-100 | L-Glutamine [10 mM] |
|  | Peprotech, Cranbury, NJ  #AF-100-15-1MG | Epidermal Growth Factor (EGF) [10 ng/mL] |
|  | Alfa Aesar, Tewksbury, MA  A1629203 | Hydrocortisone [10 µg/mL] |
|  | * | Amphotericin B [250 µg/mL] |
| C2C12 Myoblast cell line | Corning  #10017CM | DMEM HG without Sodium Pyruvate |
|  | * | 10% FBS |
|  | * | 1% P/S |
|  | * | Amphotericin B [250 µg/mL] |
| ASC52telo adipose derived stem cell line | ATCC, Manassas VA  #PCS-500-030 | Mesenchymal Stem Cell (MSC) Basal Medium |
|  | ATCC  #PCS-500-030 | 2% FBS |
|  | ATCC  #PCS-500-030 | FGF basic [5 ng/mL] |
|  | ATCC  #PCS-500-030 | FGF acidic [5ng/mL] |
|  | ATCC  #PCS-500-030 | L-Alanyl-L-Glutamine [2.4 mM] |
|  | ATCC  #PCS-500-030 | G418 [0.2 mg/mL] |
| VBAM growth media (day 0-4 of culture) | * | MCDB131 Base medium |
|  | * | 10% FBS |
|  | * | 1% P/S |
|  | * | Amphotericin B [250 µg/mL] |
|  | * | L-Glutamine [10 mM] |
|  | * | Epidermal Growth Factor (EGF) [10 ng/mL] |
|  | * | Hydrocortisone [10 µg/mL] |
|  | Fisher Chemical, Fair Lawn, NJ  #A61-100 | L-Ascorbic Acid [50 µg/mL] |
|  | TCI, Portland, OR  #H0296 | Trans-4-Hydroxy-L-proline [10 mg/L] |
|  | Fisher Bioreagents  #BP392-100 | L-Proline [40 mg/L] |
|  | Peprotech  100-20-1MG | VEGF [1 ng/mL] |
| VBAM differentiation media (day 5-40 of culture) | * | MCDB131 Base medium |
|  | * | 2% FBS |
|  | * | 1% P/S |
|  | * | Amphotericin B [250 µg/mL] |
|  | * | L-Glutamine [10 mM] |
|  | * | Epidermal Growth Factor (EGF) [10 ng/mL] |
|  | * | Hydrocortisone [10 µg/mL] |
|  | Gibco, Grand Island, NY  #41400045 | 1x Insulin-Transferrin-Selenium |
|  | * | VEGF [1 ng/mL] (day 5-14) |
|  | * | PDGF-BB [0.1 ng/mL] (day 14-40) |

*** Indicates source information was previously defined.**
