# Supplementary material for: In vitro formation and extended culture of highly metabolically active and contractile tissues
Source: PLoS One. 2023 Nov 1;18(11):e0293609. doi: 10.1371/journal.pone.0293609 (PMC10619834; doi:10.1371/journal.pone.0293609)
Supplement: S2 Table — A summary of primary and secondary antibodies used for 3D cell culture staining. (DOCX) [file pone.0293609.s007.docx]

| **Primary Antibody** | **Source** | | **Concentration** | | **Use** |
| --- | --- | --- | --- | --- | --- |
| *DyLight 554 Phalloidin* | 13054; Cell Signaling Technologies, Danvers, MA | | [1:200] | | F-actin marker |
| *DRAQ7* | 7406; Cell Signaling Technologies | | [1:100] | | Nuclear marker |
| *Myosin (Fast) mouse monoclonal* | M1570; Sigma-Aldrich | | [1:250] | | Muscle differentiation marker^1,2^ |
| *Titin mouse monoclonal* | 9 D10; Developmental Studies Hybridoma Bank, Iowa City, IA | | [1:100] | | Muscle differentiation marker^3,4^ |
| *mCherry rabbit polyclonal* | PA5-34974; Invitrogen, Rockford, IL | | [1:250] | | Label mCherry tagged HMEC1 cells |
|  | | | | | |
| **Secondary Antibody** | **Source** | | **Concentration** | | **Use** |
| *Goat Anti-Rabbit IgG DyLight™ 550 Conjugated* | 84541; Invitrogen | | [1:500] | | mCherry secondary |
| *Goat Anti-Mouse IgG, DyLight™ 488 Conjugated* | 35502; Invitrogen | | [1:500] | | Myosin or Titin secondary |
|  | | | | | |
| **BLOCKING BUFFER** (500 mL) | | | | | |
| ***Reagent*** | | ***Source*** | | ***Amount*** | |
| ddH_2_O | | - | | 450 mL | |
| 10 x PBS | | Apex Bioresearch Products, Houston, TX  #20-134 | | 50 mL | |
| Bovine Serum Albumin (BSA) | | Genesee Scientific 25-529 | | 5 g | |
| Tween 20 | | Fisher BioReagents BP337 | | 0.5 mL | |
| Cold water Fish Gelatin | | Sigma-Aldrich G7041 | | 1 g | |
| Sodium Azide (10% Sodium Azide in diH_2_O) | | Fisher Chemical  S227I | | 5 mL (0.1% final concentration) | |
