# Supplementary material for: In vitro formation and extended culture of highly metabolically active and contractile tissues
Source: PLoS One. 2023 Nov 1;18(11):e0293609. doi: 10.1371/journal.pone.0293609 (PMC10619834; doi:10.1371/journal.pone.0293609)
Supplement: S1 File — Extended experimental procedures. (DOCX) [file pone.0293609.s008.docx]

**Supplementary materials and methods**

**P-PDMS mold fabrication**

For collagen contractility studies, 20 g granulated sugar (approximately 400-500 µm in diameter) was packed into a 90 mm petri dish around a 3D printed mold (Fig 1B) [1]. Then 15 g of PDMS prepolymer was poured into the dish and cured for 48 h at 55°C. After P-PDMS was demolded from the 3D print and sugar was leached out in water changed daily for 7 d at 40°C. To verify sugar had been fully removed and to dry the P-PDMS, molds were placed in the oven at 120°C for 2 h. If browning was observed, this was taken as an indication of sugar caramelization indicating incomplete porogen removal; these molds were discarded.

Commercially available sugar cubes (1.5 cm x 1.5 cm x 1 cm) were used as a sacrificial template for 3D culture metabolic studies. It has previously been reported that sugar cube templates form molds with interconnected pores [2–4]. Sugar cubes were placed in a plastic cup and 30 g PDMS prepolymer was poured on top of them under vacuum. After 1 hr the molds were transferred to an oven and cured for 48 h at 55°C. Sugar leaching and verification were performed as described above. P-PDMS molds were cut to be 1/2 in tall with a 1/4 in diameter well at the center and adhered to glass coverslips (22 mm x 40 mm) with PDMS prepolymer (Fig 1A). Prior to PDA functionalization P-PDMS molds were rewet in an ethanol gradient. Briefly, molds were submerged in 100% EtOH for 2 hrs, followed by overnight incubation in 50% EtOH:H2O, 1 hr in 25% EtOH:H2O, and 1 hr in 100% H2O the following morning. Infiltration of water into the pores was readily visible when compared to dried molds (Fig 1A). Porosity was further confirmed by loading the center well and observing leakage to the outside.

To assess pore size in P-PDMS we thinly sliced P-PDMS formed from sugar cubes and placed it on a glass slide for imaging with a Trinocular Inverted Microscope (VWR, Radnor, PA) with a 3MP digital camera (MU300; AmScope, Irvine, CA). Representative images (n = 6) were processed in MATLAB via a median filter with a 1.30 x 1.30 µm kernel. Pores were segmented using hysteresis thresholding with empirically determined limits of 115 and 200. Maximum Feret diameter was used to quantify pore diameter. We found that P-PDMS molds had a mean pore diameter of 50.4 ± 4.01 µm.

**VBAM model fabrication**

B.I.D feedings were performed using an automated syringe pump. differentiation media was prepared in an autoclaved beaker in parallel with the MWF differentiation media. Prior to feeding, 2 pieces of tubing (0.07 in outer diameter, 18 in long, Cole-Parmer, Vernon Hills, IL) and 2 18 ga. steel blunt tip dispensing cannulas were sterilized in autoclave pouches. 90 mm tissue culture dish lids were drilled with a 1/16 in bit to create 2 holes on opposite ends for tubing insertion. Lids were sterilized in 70% ethanol prior to use. For media infusion a sterile 60 mL plastic Luer slip syringe was filled with 60 mL VBAM differentiation media and connected to an 18 ga. steel dispensing tip and one of the tubing sections. Tubing was primed with media and insert into the tissue culture dish lid and stabilized with tape. Similarly, a media withdrawal syringe was connected to the remaining dispensing tip and tubing section and insert into the dish lid, ensuring that the tubing end was flush with the bottom of the plate. The plate was primed with 13 mL of media. The media exchange system and VBAM samples were then transferred to the cell culture incubator and tubing was taped in place. Each syringe was connected to a multi-step programmable syringe pump (Chemyx; Stafford, TX). Media withdrawal occurred every 12 h and media infusion occurred every 12 hr with a 15 minute delay to ensure a fresh bolus was delivered.

**Image acquisition, tissue clearing, and volumetric quantification**

Briefly, noise was removed from the nuclei channel using a median filter with a 1.07 µm x 1.07 µm x 7.23 µm kernel and background illumination was smoothed via tophat filtering with a sphere structuring element (2.1 µm). Intensities were scaled volumetrically using a linear image adjustment, and additional noise was removed with median filtering using a 1.79 µm x 1.79 µm x 12.0 µm kernel. Nuclei were segmented using hysteresis thresholding with empirically determined limits of 75 and 85. Finally, small artifacts were removed from the binary volume via area opening. Nuclear volume fraction was defined as the sum of nuclei positive voxels in the binary volume and divided by the total voxel number.

**Polarization-sensitive optical coherence tomography**

The described PS-OCT system has a center wavelength of 1310 nm with axial and lateral resolution of 11 µm and 37 µm, respectively. The imaging depth is 2 mm in the air with a sensitivity roll-off of 13 dB over that range. The VBAM samples were submerged in 1× PBS during image acquisition to reduce specular reflection from the surface and were pinned at the ends. The sample region between the two pinned ends were scanned volumetrically in overlapping volumes with 1-2 mm overlap between the volumes. The volumes were manually aligned during post-processing. Each volume is comprised of 200 cross-sectional images which encompasses 4.5 mm length of the sample. The cross-sectional images have 1024 A-lines encompassing 1.125 mm width.

The post-processing of volumetric data was performed with MATLAB. The structural intensity images were generated with standard Fourier domain processing method [5–9] which shows the intensity of light backscattered from the samples (Figs 8A & 8E). Muscle tissue also exhibits form birefringence [10], an optical property arising from the structural anisotropy of long, parallel fibrils embedded in a medium of different refractive index. When polarized light passes through a birefringent medium, the two orthogonal polarization components of light travel at different speeds due to the difference in their refractive indices introducing a phase retardation between the two components. PS-OCT can measure this phase retardation and optic axis [11–14] which are measures of the degree of organization and orientation of the fibrous structure, respectively. We used the spectral binning algorithm [15] to measure the local phase retardation ρ (Figs 8B & 8F) and optic axis vector $(q, u, v)$. Ideally, the measured optic axis should lie on the QU-plane on a Poincaré sphere representation. However, because of the birefringence of the fiber-based imaging system, the measured optic axis vector is rotated off the QU-plane [14]. From the rotated optic axis, we obtained the relative orientation of the muscle fibers by measuring the orientation angle θ with respect to a manually defined reference angle (Figure 8C & 8G).

Nam et al. (2018) recently proposed vectorial birefringence, defined as $\vec{B}=\rho[cos\theta sin\theta]$, which adds directional contrast to the scalar birefringence used in previous studies [16–18].The vectorial birefringence is presented in HSV colormap where the H channel sets the color and the V channel sets the brightness of the color. In the vectorial birefringence images (Figs 8D & 8H), the color encodes the muscle fiber orientation $\theta$ and the brightness of the color scales with the phase retardation $\rho$. The brightness was multiplied by the intensity to exclude noise dominant pixels. The cross-sectional vectorial birefringence images were then compiled in Amira for generating 3D images of the samples (Figs 8I & 8J).

**Glucose assay**

A colorimetric glucose assay was performed according to manufacturer’s directions (10009582; Cayman Chemical, Ann Arbor, MI). Media was diluted [1:25] in assay buffer. Glucose standards and diluted samples were added to a 96 well plate in duplicate and combined with provided colorimetric enzyme mixture. The plate was incubated for 10 min at 37°C before reading on a plate reader (SpectraMax M2, Molecular Devices, San Jose, CA) at 513 nm. Output absorbances were corrected by taking the mean of each duplicate and subtracting the mean absorbance of the 0 mg/dL glucose standard. Corrected absorbances were normalized to the corrected absorbance of control MDCK media.

**References**

1. Choi S-J, Kwon T-H, Im H, Moon D-I, Baek DJ, Seol M-L, et al. A Polydimethylsiloxane (PDMS) Sponge for the Selective Absorption of Oil from Water. ACS Appl Mater Interfaces. 2011;3: 4552–4556. doi:10.1021/am201352w

2. Ren X, Lu H, Zhou JG, Chong PL-G, Yuan W, Noh M. Porous Polydimethylsiloxane as a Gas–Liquid Interface for Microfluidic Applications. Journal of Microelectromechanical Systems. 2017;26: 120–126. doi:10.1109/JMEMS.2016.2618395

3. González-Rivera J, Iglio R, Barillaro G, Duce C, Tinè MR. Structural and Thermoanalytical Characterization of 3D Porous PDMS Foam Materials: The Effect of Impurities Derived from a Sugar Templating Process. Polymers. 2018;10: 616. doi:10.3390/polym10060616

4. Li Q, Duan T, Shao J, Yu H. Fabrication method for structured porous polydimethylsiloxane (PDMS). J Mater Sci. 2018;53: 11873–11882. doi:10.1007/s10853-018-2396-z

5. Mitsui T. Dynamic Range of Optical Reflectometry with Spectral Interferometry. Jpn J Appl Phys. 1999;38: 6133. doi:10.1143/JJAP.38.6133

6. Wojtkowski M, Leitgeb R, Kowalczyk A, Bajraszewski T, Fercher AF. In vivo human retinal imaging by Fourier domain optical coherence tomography. J Biomed Opt. 2002;7: 457. doi:10.1117/1.1482379

7. Choma M, Sarunic M, Yang C, Izatt J. Sensitivity advantage of swept source and Fourier domain optical coherence tomography. Opt Express. 2003;11: 2183. doi:10.1364/OE.11.002183

8. de Boer JF, Cense B, Park BH, Pierce MC, Tearney GJ, Bouma BE. Improved signal-to-noise ratio in spectral-domain compared with time-domain optical coherence tomography. Opt Lett. 2003;28: 2067. doi:10.1364/OL.28.002067

9. Nassif N, Cense B, Hyle Park B, Yun SH, Chen TC, Bouma BE, et al. In vivo human retinal imaging by ultrahigh-speed spectral domain optical coherence tomography. Opt Lett. 2004;29: 480. doi:10.1364/OL.29.000480

10. Haskell RC, Carlson FD, Blank PS. Form birefringence of muscle. Biophysical Journal. 1989;56: 401–413. doi:10.1016/S0006-3495(89)82686-4

11. Hee MR, Huang D, Swanson EA, Fujimoto JG. Polarization-sensitive low-coherence reflectometer for birefringence characterization and ranging. J Opt Soc Am B, JOSAB. 1992;9: 903–908. doi:10.1364/JOSAB.9.000903

12. de Boer JF, Milner TE, Van Gemert MJC, Nelson JS. Two-dimensional birefringence imaging in biological tissue by polarization-sensitive optical coherence tomography. Opt Lett. 1997;22: 934. doi:10.1364/OL.22.000934

13. Park BH, Saxer C, Srinivas SM, Nelson JS, de Boer JF. In vivo burn depth determination by high-speed fiber-based polarization sensitive optical coherence tomography. J Biomed Opt. 2001;6: 474. doi:10.1117/1.1413208

14. Park BH, Pierce MC, Cense B, de Boer JF. Optic axis determination accuracy for fiber-based polarization-sensitive optical coherence tomography. Opt Lett. 2005;30: 2587. doi:10.1364/OL.30.002587

15. Villiger M, Zhang EZ, Nadkarni SK, Oh W-Y, Vakoc BJ, Bouma BE. Spectral binning for mitigation of polarization mode dispersion artifacts in catheter-based optical frequency domain imaging. Opt Express. 2013;21: 16353. doi:10.1364/OE.21.016353

16. Baumann B. Polarization Sensitive Optical Coherence Tomography: A Review of Technology and Applications. Applied Sciences. 2017;7: 474. doi:10.3390/app7050474

17. de Boer JF, Hitzenberger CK, Yasuno Y. Polarization sensitive optical coherence tomography – a review [Invited]. Biomed Opt Express. 2017;8: 1838. doi:10.1364/BOE.8.001838

18. Nam AS, Easow JM, Chico-Calero I, Villiger M, Welt J, Borschel GH, et al. Wide-Field Functional Microscopy of Peripheral Nerve Injury and Regeneration. Sci Rep. 2018;8: 14004. doi:10.1038/s41598-018-32346-w
